# Development and Validation of a Composite Programmatic Assessment Tool for HIV Therapy

**DOI:** 10.1371/journal.pone.0047859

**Published:** 2012-11-19

**Authors:** Viviane D. Lima, Adrian Le, Bohdan Nosyk, Rolando Barrios, Benita Yip, Robert S. Hogg, P. Richard Harrigan, Julio S. G. Montaner

**Affiliations:** 1 British Columbia Centre for Excellence in HIV/AIDS, St. Paul's Hospital, Vancouver, British Columbia, Canada; 2 Division of AIDS, Department of Medicine, Faculty of Medicine, University of British Columbia, Vancouver, British Columbia, Canada; 3 Faculty of Medicine, University of British Columbia, Vancouver, British Columbia, Canada; 4 Faculty of Health Sciences, Simon Fraser University, Burnaby, British Columbia, Canada; Indiana University, United States of America

## Abstract

**Background:**

We developed and validated a new and simple metric, the Programmatic Compliance Score (PCS), based on the IAS-USA antiretroviral therapy management guidelines for HIV-infected adults, as a predictor of all-cause mortality, at a program-wide level. We hypothesized that non-compliance would be associated with the highest probability of mortality.

**Methods and Findings:**

3543 antiretroviral-naive HIV-infected patients aged ≥19 years who initiated antiretroviral therapy between January 1, 2000 and August 31, 2009 in British Columbia (BC), Canada, were followed until August 31, 2010. The PCS is composed by six non-performance indicators based on the IAS-USA guidelines: (1) having <3 CD4 count tests in the first year after starting antiretroviral therapy; (2) having <3 plasma viral load tests in the first year after starting antiretroviral therapy; (3) not having drug resistance testing done prior to starting antiretroviral therapy; (4) starting on a non-recommended antiretroviral therapy regimen; (5) starting therapy with CD4 <200 cells/mm^3^; and (6) not achieving viral suppression within 6 months since antiretroviral therapy initiation. The sum of these six indicators was used to develop the PCS score - higher score indicates poorer performance. The main outcome was all-cause mortality. Each PCS component was independently associated with mortality. In the mortality analysis, the odds ratio (OR) for PCS ≥4 versus 0 was 22.37 (95% CI 10.46–47.84).

**Conclusions:**

PCS was strongly associated with all-cause mortality. These results lend independent validation to the IAS-USA treatment guidelines for HIV-infected adults. Further efforts are warranted to enhance the PCS as a means to further improve clinical outcomes. These should be specifically evaluated and targeted at healthcare providers and patients.

## Introduction

HIV treatment has evolved tremendously since the advent of highly active antiretroviral therapy (HAART) in 1996 [1]–[9]. The goal of HAART is to decrease HIV-related morbidity and mortality through sustained full suppression of viral replication (i.e. plasma viral load <50 copies/mL). More recently, HAART has also been recognized as a highly effective strategy to prevent HIV transmission [10]. Definitive confirmation of the efficacy of HIV treatment as prevention was provided by the recent results of the HPTN 052 trial [11]. In this study, immediate use of HAART was shown to decrease HIV transmission by 96%. This has generated a renewed enthusiasm in the global roll out of HAART [12], [13]. However, it is still not clear which metrics, at the individual and the population levels, should be used to monitor and evaluate the impact of this powerful intervention.

HIV treatment guidelines provide evidence-based standards aimed to optimize the management of HIV infected individuals. However, for a variety of reasons, not all patients are fully adherent to these guidelines. Here, we developed and validated a composite metric, the Programmatic Compliance Score, to assess the impact of non-compliance with HIV treatment guidelines on all-cause mortality, among antiretroviral therapy-treated HIV-infected individuals within a fully subsidized, population-based antiretroviral therapy program. We hypothesized that non-compliance would be associated with the highest chance of dying prematurely.

## Methods

### Ethical Approval

The Centre's HIV/AIDS Drug Treatment program has received ethical approval from the University of British Columbia Ethics Review Committee at its St. Paul's Hospital site. The program also conforms with the province's Freedom of Information and Protection of Privacy Act.

### HIV Patients on Treatment in British Columbia

This study was conducted using population data from the British Columbia (BC) Centre for Excellence in HIV/AIDS (the Centre) [7]. The Centre's guidelines have remained consistent with recommendations of the IAS-USA since 1996 and up to the latest guidelines in 2010 [1]–[9]. The details of this program have been described elsewhere [14]. In BC, medical and laboratory monitoring, including specialized tests such as CD4 cell counts, plasma viral load, genotypic resistance testing are all fully subsidized. The Centre has received ethical approval from the University of British Columbia Ethics Review Committee at the St Paul's Hospital site.

### Study Population

Eligible study participants were ≥19 years old, naïve to antiretroviral therapy when they started treatment between January 1, 2000 and August 31, 2009. These patients were followed until death due to any cause, or if alive, until the last contact date or August 31, 2010, whichever came first. Finally, to be eligible for this analysis, participants were required to have at least one baseline CD4 cell count and a plasma viral load measurement available within six months prior to the antiretroviral starting date.

### Laboratory Data

All plasma viral load measurements in BC are centrally done at the St Paul's Hospital virology laboratory. The quantification range of plasma viral load assays has evolved over time, as previously described. Thus, for analytical purposes, we truncated our measurements to range from <50 to >100,000 copies/mL. CD4 cell counts are measured by flow cytometry, followed by fluorescent monoclonal antibody analysis (Beckman Coulter, Inc., Mississauga, Ontario, Canada). The CD4 data come from different laboratories across BC, and, in our database, we capture >80% of all CD4 tests done in the province. HIV genotypic resistance testing is performed centrally at the Centre's laboratory. Samples have been assigned to one of four resistance categories based on a modification of the 2011 IAS-USA list of mutations [15].

### Antiretroviral Regimen

The recommended antiretroviral therapy regimens have changed since 2000, based on the BC guidelines for treating HIV-positive adults, as in the IAS-USA guidelines [4]–[9]. Therefore, we developed rules to classify regimes as contemporary appropriate or not, as described in the Supplementary text.

### The Programmatic Compliance Score

We developed six performance indicators based on the IAS-USA guideline recommendations during 2000–2010 [4]–[9]: (1) Having <3 (coded as 1) or ≥3 (coded as 0) CD4 cell count measurements in the first year after starting antiretroviral therapy; (2) Having <3 (coded as 1) or ≥3 (coded as 0) plasma viral load measurements in the first year after starting antiretroviral therapy; (3) Having a genotypic resistance test performed (coded as 0) or not (coded as 1) at baseline; (4) Initiating antiretroviral therapy with baseline CD4 cell count with <200 cells/mm^3^ (coded as 1) or ≥200 cells/mm^3^ (coded as 0); (5) Initiating antiretroviral therapy on a combination regimen recommended by contemporary guidelines (coded as 0) or not (coded as 1); and (6) Achieving viral suppression within 6 months of initiating antiretroviral therapy (coded as 0) or not (coded as 1). Viral suppression was defined by two consecutive plasma viral loads <50 copies/mL. Our main measure of exposure, the programmatic compliance score (PCS), was then obtained by adding the values for indicators 1 to 6, which provided a range from 0 (least compliance) to 6 (most non-compliance).

### Outcome Measure and Statistical Analyses

The primary outcome in this study was all-cause mortality. Deaths occurring during the follow-up period were identified on a continuous basis through record linkages carried out with the BC Division of Vital Statistics and enhanced by direct physician reports to the program.

Further, we considered the following baseline explanatory variables: age, gender, history of injection drug use (IDU), year of antiretroviral therapy initiation, plasma viral load, follow-up time in months and place of residence at the start of antiretroviral therapy. Place of residence was used to control for the heterogeneity in patient treatment access, care and socio-demographic factors not previously defined. We also considered adherence to antiretroviral therapy measured at 12 months from antiretroviral therapy initiation, since the calculation of adherence at 6 months in our database can yield less precise estimates. Adherence was estimated by dividing the number of months of medications dispensed by the number of months of follow-up. We have previously shown that this measure of adherence is independently associated with HIV viral suppression and survival [16]–[18]. Adherence was categorized as either <95% or ≥95%.

We run two sets of analysis. The first set included data from patients who have started antiretroviral therapy between 2000 and 2009. The second analysis, recognizing that some of the variables were not collected systematically before the year 2006, we restricted our analysis from patients who have started antiretroviral therapy between 2006 and 2009.

In bivariable analyses, categorical variables were compared using the Fisher's exact test, and continuous variables were compared using the Wilcoxon rank-sum test. The mortality probability model was built in terms of finding the best predictive probabilistic model of mortality, using logistic regression [19]. First, we drew a random sample without replacement from the original data, splitting the original data in half. One half was used as a training dataset, in which we built the multivariable logistic model and assessed its fit by calculating the area under the receiver operating characteristic curve. This area was used to assess the model's ability to discriminate between those who died versus those who did not. A backward stepwise technique was used in the selection of covariates to build this model. The selection of variables was based on two criteria: Akaike Information Criterion (AIC) and Type III p-values. These two criteria balance the model choice by finding the best explanatory model (Type III p-values based on the Type III Sum of Squares, with lower p-values indicate more significance) and at the same time a model with the best goodness-of-fit statistic (AIC – lower values indicate better fit). At each step of this process, the AIC value and the Type III p-values of each variable were recorded, and the variable with the highest Type III p-value was dropped, until there are no more variables left. The final model has the lowest AIC. The second half of the data was used to assess whether the predictions based on the coefficients obtained from the analysis on the training dataset were valid or not. For goodness of fit, we calculated the mean squared error, the mean absolute error and the Wilcoxon rank-sum test. Kaplan-Meier method with log-rank test was used for the comparison of the unadjusted survival rates. All analyses were performed using SAS software version 9.2 (SAS, Cary, NC).

## Results

### Cohort Characteristics

A total of 3543 antiretroviral naïve adults (79% males) were eligible to participate in this study. At baseline, the median age was 42 years (25^th^ to 75^th^ percentile range [Q1–Q3]: 35–48 years), CD4 cell count was 190 cells/mm^3^ (Q1–Q3: 100–280 cells/mm^3^), and plasma viral load was 4.9 log_10_ copies/mL (Q1–Q3: 4.4–5.0 log_10_ copies/mL). The median follow-up was 44 months (Q1–Q3: 22–77 months). Of these patients, 39% had a history of IDU, 52% started antiretroviral therapy before 2006, and 63% were more than 95% adherent during the first year of follow-up.

### Programmatic Compliance Score (PCS)

**Figure 1:**
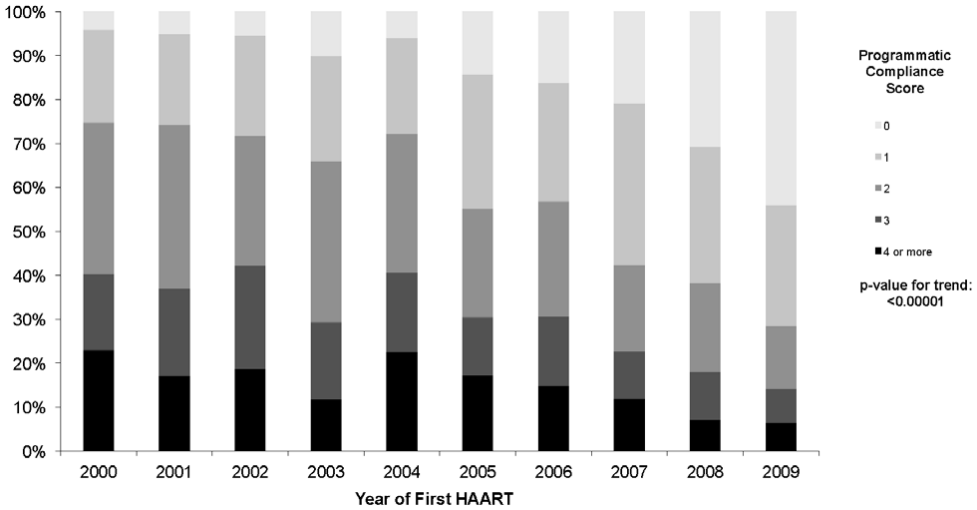
Distribution of the programmatic compliance score over time.

At baseline, 42% of patients did not receive a genotypic resistance test before therapy initiation, 11% of patients received a non-recommended antiretroviral therapy regimen, and 52% of patients had a baseline CD4 count <200 cells/mm^3^. During the first year of follow-up, 23% of patients had fewer than 3 CD4 count tests done, 16% had <3 plasma viral load tests done, and 46% did not achieve viral suppression within 6 months of beginning treatment. After several exploratory analyses, we decided to group the PCS as 0, 1, 2, 3 and 4 or more. The distribution of PCS was, then: 16% for a 0 score, 26% for a score of 1, 27% for a score of 2, 16% for a score of 3 and 14% for scores of 4 or more. Figure 1 shows the distribution of PCS over time, and as we moved from 2000 to 2009, individuals starting antiretroviral therapy on the later years were more likely to have a PCS score of 0 (p-value for trend <0.0001).

### Predictive Mortality Probability Model (Follow-Up from 2000 to 2010)

At the end of follow-up, 499 (14%) deaths were recorded, producing an overall crude death rate of 33 per 1000 person-years. Table 1 shows the bivariable association of our main exposure, indicators and baseline factors with all cause mortality. Those who have died were more likely to be older (44 versus 41 years; p-value<0.0001), to have a shorter follow-up (23 versus 47 months; p-value<0.0001), to have a history of IDU (18% versus 11%; p-value<0.0001), to have adherence <95% during the first year on antiretroviral therapy (21% versus 10%; p-value<0.0001), and to have started antiretroviral therapy during 2000–2005 (21% versus 7% p-value<0.0001). All six indicators were strongly associated with all cause mortality, and therefore, those with a PCS ≥4 were more likely to have died in this study (38% versus 20%, 12%, 7% and 2% for scores 3, 2, 1 and 0, respectively; p-value<0.0001).

**Table 1:** Descriptive statistics by mortality status at the end of follow-up.

	Deceased at the End of Follow-up		
List of Variables	No	Yes	p-value
	N = 3044	N = 499	
**Programmatic Compliance Score**			
0	565 (98%)	13 (2%)	<0.0001
1	868 (93%)	68 (7%)	
2	845 (88%)	111 (12%)	
3	449 (80%)	114 (20%)	
4 or more	317 (62%)	193 (38%)	
**Age**			
Median	41	44	<0.0001
Q1–Q3	35–48	37–51	
**Follow-up (in months)**			
Median	47	23	<0.0001
Q1–Q3	25–82	6–48	
**Gender**			
Male	2431 (86%)	385 (14%)	0.1692
Female	613 (84%)	114 (16%)	
**Injection drug use history**			
No	1918 (89%)	248 (11%)	<0.0001
Yes	1126 (82%)	251 (18%)	
**Adherence during first year**			
≥95%	2008 (90%)	216 (10%)	<0.0001
<95%	1036 (78%)	283 (21%)	
**Year of first ARV**			
2000–2005	1455 (79%)	381 (21%)	<0.0001
2006–2010	1589 (93%)	118 (7%)	
**Number of CD4 cell count measurements (1st year)**			
≥3	2412 (89%)	309 (11%)	<0.0001
<3	632 (77%)	190 (23%)	
**Number of plasma HIV-1 RNA level measurements (1st year)**			
≥3	2665 (90%)	303 (10%)	<0.0001
<3	379 (66%)	196 (34%)	
**Baseline resistance test**			
Yes	1871 (91%)	184 (9%)	<0.0001
No	1173 (79%)	315 (21%)	
**Baseline CD4 cell count (cells/mm3)**			
≥200	1535 (91%)	157 (9%)	<0.0001
<200	1509 (81%)	342 (18%)	
**Recommended HAART regimen**			
Yes	2718 (87%)	422 (13%)	<0.0001
No	326 (81%)	77 (19%)	
**Suppression at 6 month**			
Yes	1789 (93%)	127 (7%)	<0.0001
No	1255 (77%)	372 (23%)	
**Health Region**			
Vancouver Coastal HA - City Center	1961 (88%)	273 (12%)	<0.0001
Vancouver Coastal HA - DTES	196 (78%)	55 (22%)	
Vancouver Coastal HA - Other	265 (90%)	31 (10%)	
Interior HA	118 (83%)	25 (17%)	
Fraser HA	170 (80%)	42 (20%)	
Vancouver Island HA	266 (82%)	60 (18%)	
Northern HA	68 (84%)	13 (16%)	

Notes: HA: Health Authority, Q1: 25th percentile; Q3: 75th percentile.

Table 2 shows the Kaplan Meier estimates for the mortality probability by levels of the PCS score. As expected, those individuals with a PCS ≥4 were at a much higher probability of mortality throughout the study period (log-rank test p-value<0.0001), with the crude mortality estimate ranging from 0% (SE ±0%) for PCS = 0 to 20.7% (SE ±1.8%) for PCS ≥4 at 6 months to 1.1% (SE ±0.5%) for PCS = 0 to 30.2% (SE ±2.2%) for PCS ≥4 at 30 months. To predict the probability of mortality for each patient in our study, we present the coefficients in Table 3. The measures used to assess the goodness of fit of our predictive model indicated that we have obtained a really well fitted model. Thus, the odds ratio (OR) for PCS from 1, 2, 3, 4 or more versus 0 were, respectively, 3.81 (95% CI 1.73–8.42), 7.97 (95% CI 3.70–17.18), 11.51 (95% CI 5.28–25.08), and 22.37 (95% CI 10.46–47.84). We were also interested in assessing the importance of each component in the PCS score on the probability of mortality and the strongest influence related to failing to suppress plasma viral load at 6 months (OR 4.25; 95% CI 3.15–5.75; area under the curve 0.668), having <3 plasma viral load tests during the first year (OR: 4.68; 95%CI 3.49–6.28; area under the curve 0.638) and having no resistance test done at baseline (OR: 2.40; 95%CI 1.83–3.15; area under the curve 0.608).

**Table 2:** Kaplan Meier estimates for the probability of mortality by the levels of the programmatic compliance score.

List of Variables	Follow-up				Log-rank Test
	12 months	18 months	24 months	30 months	p-value
**Number of CD4 cell count measurements (1st year)**					
≥3	2.5% (±0.3%)	3.9% (±0.4%)	4.8% (±0.4%)	6.1% (±0.5%)	<0.0001
<3	12.9% (±1.1%)	14.5% (±1.2%)	15.8% (±1.3%)	17.0% (±1.3%)	
**Number of plasma HIV-1 RNA level measurements (1st year)**					
≥3	1.9% (±0.3%)	3.1% (±0.3%)	4.1% (±0.4%)	5.2% (±0.4%)	<0.0001
<3	20.4% (±1.6%)	23.1% (±1.8%)	24.6% (±1.8%)	26.4% (±1.9%)	
**Baseline resistance test**					
Yes	3.6% (±0.4%)	4.4% (±0.5%)	5.0% (±0.5%)	5.9% (±0.5%)	<0.0001
No	7.2% (±0.7%)	9.4% (±0.8%)	10.9% (±0.8%)	12.6% (±0.9%)	
**Baseline CD4 cell count (cells/mm3)**					
≥200	2.2% (±0.4%)	3.1% (±0.4%)	3.9% (±0.5%)	4.9% (±0.6%)	<0.0001
<200	7.8% (±0.6%)	9.8% (±0.7%)	10.9% (±0.7%)	12.4% (±0.8%)	
**Recommended cART regimen**					
Yes	3.2% (±0.5%)	4.5% (±0.5%)	4.6% (±0.6%)	5.2% (±0.7%)	0.0125
No	6.2% (±0.5%)	8.0% (±0.6%)	9.2% (±0.6%)	10.7% (±0.7%)	
**Suppression at 6 month**					
Yes	0.9% (±0.2%)	1.6% (±0.3%)	2.1% (±0.3%)	2.8% (±0.4%)	<0.0001
No	9.9% (±0.7%)	12.2% (±0.8%)	13.8% (±0.9%)	15.7% (±0.9%)	
**Programmatic Compliance Score**					
0	0.4% (±0.3%)	0.8% (±0.4%)	0.8% (±0.4%)	1.1% (±0.5%)	
1	0.7% (±0.3%)	1.4% (±0.4%)	1.9% (±0.5%)	2.6% (±0.6%)	
2	1.8% (±0.4%)	3.0% (±0.6%)	4.0% (±0.7%)	5.0% (±0.7%)	<0.0001
3	7.6% (±1.1%)	10.3% (±1.1%)	12.3% (±1.4%)	14.8% (±1.6%)	
4 or more	23.6% (±1.9%)	26.7% (±2.0%)	28.4% (±2.1%)	30.2% (±2.3%)	

**Table 3:** Results from the predictive model for the probability of mortality based on the programmatic compliance score.

Variables Necessary to Estimate the Probability of Mortality	Maximum Likelihood Estimates			
	Coefficient	Standard Deviation	Odds Ratio (95% Confidence Interval)	Type III P-value
Programmatic Compliance Score				
0	0.0	0.0	1(-)	<0.0001
1	1.3387	0.4042	3.81 (1.73–8.42)	
2	2.0761	0.3916	7.97 (3.70–17.18)	
3	2.4434	0.3974	11.51 (5.28–25.08)	
4 or more	3.1079	0.3878	22.37 (10.46–47.84)	
**Also Adjust Model for:**				
Intercept	−4.4132	0.5039		<0.0001
Age (in years)	0.0324	0.00742	1.03 (1.02–1.05)	<0.0001
History of Injection Drug Use (Yes:1; No: 0)	0.5298	0.153	1.70 (1.26–2.29)	0.0005
Follow-up time in Months	−0.0242	0.00265	0.98 (0.97–0.98)	<0.0001

Goodness-of-fit	Training Dataset
Akaike Information Criterion	1185.551
Area under the Receiver Operating Curve	0.801

Goodness-of-fit	Validation Dataset
Mean Squared Error	0.274
Mean Absolute Error	0.361
Wilcoxon Rank Sum Test p-value	<0.0001

### Sensitivity Analysis

This analysis was restricted to the individuals who have started antiretroviral therapy between 2006 and 2009. In total, we observed 118 deaths (7%), producing an overall crude death rate of 30 per 1000 person-years. Table 4A shows the association of each component of the PCS score and all cause-mortality. Differently from the original analysis, the top three PCS components most influential on the probability of mortality were having <3 plasma viral load tests during the first year (OR: 7.54; 95%CI 5.10–11.16; area under the curve 0.691), failing to suppress plasma viral load at 6 months (OR: 4.66; 95%CI 3.04–7.14; area under the curve 0.680) and starting on a non-recommended antiretroviral therapy (OR: 3.78; 95%CI 2.49–5.75; area under the curve 0.657). The multivariable model fitted for this analysis aimed at finding whether PCS explains the risk of mortality, while adjusting for the same covariates as in the case of the original analysis (Table 4B). The OR for PCS from 1, 2, 3, 4 or more versus 0 were, respectively, 3.02 (95% CI 1.16–7.89), 5.01 (95% CI 1.92–13.06), 9.02 (95% CI 3.44–23.64), and 15.77 (95% CI 6.28–39.61).

**Table 4:** Relationship between the programmatic compliance score and mortality.

(A)		
	Bivariable Analysis	
List of Variables	Odds Ratio (95% Confidence Interval)	Area Under the Curve
**Number of CD4 cell count measurements (1st year)**		
≥3	1(-)	0.649
<3	3.87 (2.65–5.67)	
**Number of plasma HIV-1 RNA level measurements (1st year)**		
≥3	1(-)	0.691
<3	7.54 (5.10–11.16)	
**Baseline resistance test**		
Yes	1(-)	0.594
No	2.49 (1.69–3.67)	
**Baseline CD4 cell count (cells/mm^3^)**		
≥200	1(-)	0.527
<200	1.85 (1.04–3.28)	
**Recommended HAART regimen**		
Yes	1(-)	0.657
No	3.78 (2.49–5.75)	
**Suppression at 6 month**		
Yes	1(-)	0.680
No	4.66 (3.04–7.14)	

(B)		
Model for the Probability of Mortality	Odds Ratio (95% Confidence Interval)	Type III P-value
Programmatic Compliance Score		
0	1(-)	
1	3.02 (1.16–7.89)	
2	5.01 (1.92–13.06)	<0.0001
3	9.02 (3.44–23.64)	
4 or more	15.77 (6.28–39.61)	
**Also Adjust Model for:**		
Age (in years)	1.07 (1.05–1.09)	<0.0001
History of Injection Drug Use (Yes:1; No: 0)	2.35 (1.48–3.74)	0.0003
Follow-up time in Months	0.91 (0.89–0.93)	<0.0001

(A) Bivariable associations between each of programmatic compliance score components and mortality for individuals who started antiretroviral therapy between 2006 and 2009. (B) Results from the multivariable explanatory model for the probability of mortality based on the programmatic compliance score for individuals who started antiretroviral therapy between 2006 and 2009. Area Under the Curve: 0.896.

## Discussion

Using various indicators of non-compliance to treatment guidelines, we developed a simple and highly predictive metric, the Programmatic Compliance Score or PCS, to predict the probability of mortality among HIV-positive individuals starting naïve on antiretroviral therapy. We found that individuals who had a PCS score 4 or higher had a mortality probability 22 times higher than those individuals with PCS score 0. The sensitivity analysis also confirmed these results. The PCS, therefore, may serve as a simple but powerful proxy for the performance of the program as a whole in achieving execution of its accepted guidelines. The PCS incorporates the overall effects of the decisions and social situations of patients, physicians and others in whether or not treatment guidelines are actually implemented.

It is noteworthy and surprising that indicators associated with plasma viral load and resistance were far more impactful than suboptimal CD4 cell count at the start of therapy with respect to the probability of mortality. Low CD4 cell count at the start of antiretroviral therapy has been shown by our group and by others to be highly predictive of adverse treatment outcomes [20]–[22]. Closer monitoring of patients during their first year on antiretroviral therapy, especially plasma viral load, can increase the chances of these individuals to fully benefit from this life-saving therapy at the short- and long terms. The reasons for poor patient monitoring practices are not clear, especially in an environment in which HIV treatment, care and laboratory monitoring are fully subsidized. Further efforts are warranted to explore possible reasons for this at the health care provider and patient levels. This in turn may assist in the development of specific strategies to enhance the PCS as a means to further improve clinical outcomes. These should be specifically evaluated and targeted to health care providers as well as HIV infected clients. Furthermore, our results provide important clues on how to develop effective strategies to improve HIV associated health outcomes not only in BC, but around the world.

There are several features of our study that should be highlighted. First, this study was based on patients who were naïve to antiretroviral therapy, thus our results were not confounded by previous therapy use. Secondly, despite the potential limitations of using pharmacy refill-based adherence as a surrogate marker of actual pill taking, we have previously shown that this measure of adherence is independently associated with different disease outcomes [16]–[18]. Thirdly, delayed reporting of deaths or incomplete data collection are not likely an issue with this analysis, since all deaths were reported within three months of death through active follow-up with physicians and hospitals and regular linkages to BC Vital Statistics Agency. Fourthly, even though the guidelines prior to 2006 did not explicitly say that baseline resistance should be done prior to starting antiretroviral therapy, we decided to include this indicator in our PCS score because transmitted drug resistance has always been one of the factors that affect future disease outcomes. The PCS score is not an indicator to penalize healthcare providers, but it serves to identify areas in our treatment program that should be improved. It is important to look at temporal trends (2000–2009) so we can identify which indicators have improved and those that have not. Due to missing data in some of the indicators present in the PCS score for patients starting antiretroviral therapy during 2000–2005, we re-run the analysis including only data from patients who started antiretroviral therapy after the year 2006. Baseline resistance testing continued to be an important indicator of programmatic compliance. Fifthly, we acknowledge that the model only uses information collected during the first year on therapy, and not information on the subsequent years to predict the risk of mortality. The first year on antiretroviral therapy is really important in a patient's treatment history. If these individuals are not properly managed in the first year, we believe that it is likely that they will not be managed properly thereafter. Thus, the PCS score was developed so that we want to catch individuals with high risk of mortality due to improper disease management in the beginning of treatment, and we want to identify the areas in which our program that can be improved. Sixthly, one of the indicators in our PCS score is baseline CD4 cell count. Some may argue that is not a measure of non-compliance, but rather a biological measure of disease severity. Based on the cascade of care framework, individuals who have tested positive for HIV should be monitored from the time they get their positive test result until the time they become eligible to receive antiretroviral therapy. In BC, as in many other places around the world, over the years, we have lost many patients in this period of time. These individuals lost to follow-up after they test positive have most often showed up in our emergence departments with, sometimes, a CD4 cell count almost close to zero. This is completely unacceptable. Thus, CD4 cell count at antiretroviral presentation is an indicator of the performance of our system, and not only a biological indicator. Seventhly, although we adjusted our analyses for several demographic and clinical characteristics, as in all observational studies unmeasured differences may exist among study populations, and for this reason, our findings should be interpreted cautiously. Finally, given that this study was conducted at the population-level within a fully subsidized medical system where antiretroviral therapy as well as medical and laboratory monitoring are free of charge to all participants, we are confident that our results are less likely to be biased by direct financial limitations to access to health services, a frequent confounder in cohort and population based studies.

In summary, the Programmatic Compliance Score or PCS metric is highly predictive of all-cause mortality, among HIV infected adults starting antiretroviral therapy. Our results show that individuals with sub-optimal PCS compliance are at a very high probability of premature morbidity and mortality. It is important to mention that the requirement for having a baseline genotypic testing before starting antiretroviral therapy was not explicitly stated in the guidelines prior to 2006, and only emphasized in the guidelines after 2006. However, given that the efficacy of antiretroviral therapy is directly related to having a fully functional regimen, there is no reason for failing to order such an important test, given that it is free for anyone starting antiretroviral therapy in BC. Thus, while these results do not allow us to establish a causal relationship regarding the association between our new metric and survival, this metric highlights the importance of adherence to treatment and monitoring guidelines during the first year on antiretroviral therapy. Despite improvement in the PCS within our cohort over time, it is clear that there is still substantial room for improvement. It should be emphasized that minimizing the occurrence of these non-compliance practices is not solely dependent on the healthcare provider. Health administrative bodies can improve compliance outcomes through regular surveillance and ongoing physician training. Further, the individual patient ultimately bears responsibility for his/her own health; the implications of poor compliance to treatment need to be communicated at the time of initiation. Furthermore, our results provide important clues on how to develop effective strategies to improve HIV associated health outcomes not only in BC, but around the world. Finally, our results also lend independent validation to the most recent IAS-USA antiretroviral therapy management guidelines for HIV infected adults.

## Supporting Information

Text S1 **Appropriate Regimens based on the BC guidelines for treating HIV-positive adults between 2000 and 2010.**
(DOCX)
