# Supplementary material for: Development and Validation of a Composite Programmatic Assessment Tool for HIV Therapy
Source: PLoS One. 2012 Nov 19;7(11):e47859. doi: 10.1371/journal.pone.0047859 (PMC3501505; doi:10.1371/journal.pone.0047859)
Supplement: Text S1 — Appropriate Regimens based on the BC guidelines for treating HIV-positive adults between 2000 and 2010. (DOCX) [file pone.0047859.s001.docx]

**Supporting Information**

**Text S1.** Appropriate Regimens based on the BC guidelines for treating HIV-positive adults between 2000 and 2010.

**Years 2000-2003**

- Two comparable nucleosides, or a nucleoside and a nucleotide reverse transcriptase inhibitor plus either (1) a non-nucleoside reverse transcriptase inhibitor, or (2) a protease inhibitor boosted with ritonavir, or (3) a single protease inhibitor;
- Three nucleoside reverse transcriptase inhibitors if:
  - - zidovudine + lamivudine + abacavir; or
    - zidovudine + didanosine + lamivudine; or
    - lamivudine + stavudine + abacavir.

**Years 2004-2005**

- Two nucleoside reverse transcriptase inhibitors if:
  - - zidovudine or tenofovir + (lamivudine or emtricitabine); or
    - emtricitabine + didanosine.
- Plus either:
  - - efavirenz or nevirapine; or
    - lopinavir, atazanavir, indinavir or saquinavir boosted with ritonavir.
    - nelfinavir

**Years 2006-2008**

- Two nucleoside reverse transcriptase inhibitors if:
  - - emtricitabine + tenofovir; or
    - (abacavir or zidovudine) + lamivudine; or
    - tenofovir + lamivudine
- Plus either:
  - - efavirenz or nevirapine; or
    - lopinavir, atazanavir, amprenavir, fosamprenavir or darunavir boosted with ritonavir.

**Years 2008-2009**

- Two nucleoside reverse transcriptase inhibitors if:
  - - emtricitabine + tenofovir; or
    - abacavir + lamivudine
- Plus either:
  - - efavirenz or nevirapine; or
    - lopinavir, atazanavir, amprenavir, fosamprenavir or darunavir boosted with ritonavir; or
    - maraviroc
